# Precautionary Circulars State Board of Health

**Published:** 1890-08

**Authors:** 


### Precautionary Circulars of The State Board of Health.

These consist of a series of small pamphlets intended for heads of families, containing concise directions for care of the health of the members of families.

The budget before us consists of, "Precautions against Consumption," already noticed in our pages (see May number, page 237) ; "School Hygiene," giving concise and admirable directions concerning ventilation, heating, hours of study, exercise, wet clothing, cleanliness, water supply, care of eyes, etc. The third of the series is entitled "Precautions against Typhoid Fever," and gives full information for preventing the invasion of typhoid and for checking its spread if it have already gained an entrance. "Precautions against Scarlet Fever," and "Precautions against Contagious and Infectious Diseases" in general are also very important ; and, finally, there is a special circular concerning the care of infants. All these instructive publications are inclosed in a neat case and should be in the hands of all heads of families and carefully preserved for future reference. They will be forwarded to any address on receipt of a two-cent stamp, by applying to the Secretary of the State Board of Health,

Dr. Benjamin Lee,
1532 Pine Street, Philadelphia, Pa.

